# Knitting polycyclic aromatic hydrocarbon-based microporous organic polymers for efficient CO_2_ capture†

**DOI:** 10.1039/c8ra01332b

**Published:** 2018-03-13

**Authors:** Shuangshuang Hou, Shaolei Wang, Xuejun Long, Bien Tan

**Affiliations:** Key Laboratory for Large-Format Battery Materials and System, Ministry of Education, Hubei Key Laboratory of Material Chemistry and Service Failure, School of Chemistry and Chemical Engineering, Huazhong University of Science and Technology Wuhan 430074 P. R. China bien.tan@mail.hust.edu.cn +86 27 87558172 +86 27 87558172; Engineering Research Center for Clean Production of Textile Printing and Dyeing, Ministry of Education, Wuhan Textile University Wuhan 430073 P. R. China

## Abstract

In order to achieve efficient CO_2_ capture, four novel microporous organic polymers, based on distinct polycyclic aromatic hydrocarbons such as fluoranthene, binaphthalene, naphthalene and phenanthrene, were successfully prepared by the solvent knitting method. N_2_ sorption isotherms indicate that these polymers are predominately microporous with ultrahigh BET surface area *i.e.*, 1788 m^2^ g^−1^ for fluoranthene-based Polymer 1, 1702 m^2^ g^−1^ for binaphthalene-based Polymer 2 and objective CO_2_ uptake capacity of 24.79 wt% and 20.19 wt% (273.15 K/1.00 bar) respectively. While compared with the former two polymers, though 1227 m^2^ g^−1^ and 978 m^2^ g^−1^ are moderate in surface area, however the naphthalene-based Polymer 3 and phenanthrene-based Polymer 4 still exhibit CO_2_ adsorption of up to 17.44 wt% and 18.15 wt% respectively under the similar conditions. Moreover, the H_2_ storage and CH_4_ adsorption in these polymers can be 2.20 wt% (77.3 K/1.13 bar) and 2.79 wt% (273.15 K/1.00 bar). More significantly, the electron-rich PAHs are proved to be new building blocks that provide a wealth of chances to produce hypercrosslinked polymers with efficient gas adsorption capacity, which are greatly influenced by the porous nature of polymers. Given the merits including mild reaction conditions, low cost, high surface area, impressive gas absorption performance, high thermal stability, these polymers are considered to be promising candidates for CO_2_ capture and energy storage under more practical conditions.

## Abstract 2

In order to achieve efficient CO_2_ capture, four novel microporous organic polymers, based on distinct polycyclic aromatic hydrocarbons, were successfully prepared by the solvent knitting method.

## Graphical abstract

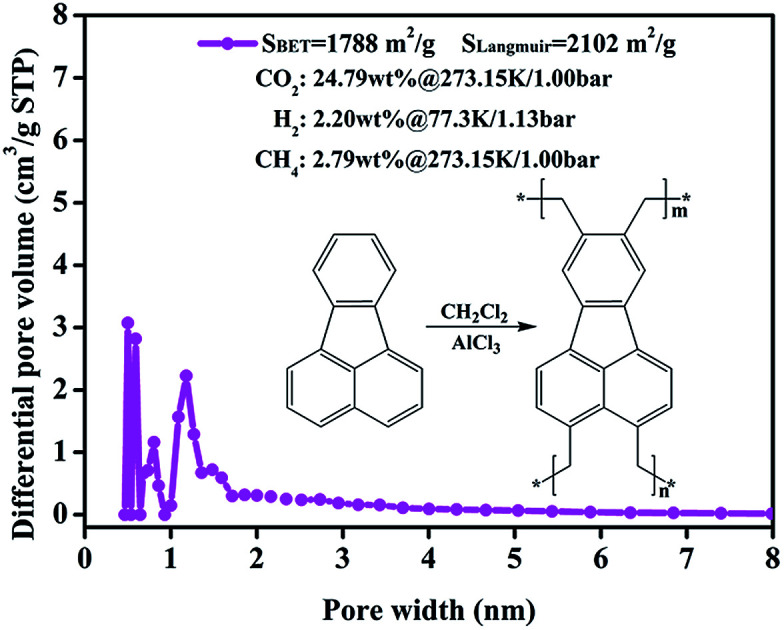

## Introduction

Deemed as a dominating contributor to the worldwide greenhouse effect, CO_2_ has been receiving a great deal of attention for years. As a result of burning fossil fuels including coal, oil and gas to meet the need for energy, CO_2_ is being released to the atmosphere,^1–4^ and the increasing atmospheric CO_2_ concentration has witnessed a fearful impact on the environment such as the continuous rise of water-level in sea, the growing number of ocean storms and floods as well as many other extreme weather events.^5^ Anxiety of how to effectively achieve CO_2_ capture and storage has been on rise in the scientific community, which resulted in considerable interest to develop new functional materials and even technologies to address this matter on a global scale.^6–11^

Emerged in the past few decades, hypercrosslinked polymers (HCPs) with many interesting intrinsic features such as large specific surface area, low skeleton density and narrow pore size distribution have exhibited great potential for CO_2_ uptake.^12^ Bearing the motivations of diverse arts, many relative contributions upon the capture of CO_2_ in HCPs have been successively unfolded. For example, Cooper *et al.* reported a high surface area network of 1015 m^2^ g^−1^ with CO_2_ capture capacity up to 3.96 mmol g^−1^ at 273.15 K/1.00 bar.^13^ Our group demonstrated a cobalt coordinated polymer with a high BET surface area 1360 m^2^ g^−1^ possessing CO_2_ adsorption of 21.39 wt% at 273.15 K/1.00 bar.^14^ Jiang and coworkers prepared a nitrogen-rich material (FCDTPA) with surface area of 871 m^2^ g^−1^ and CO_2_ uptake ability up to 2.83 mmol g^−1^ at 273.15 K/1.00 bar.^15^

Usually, a careful selection of building blocks is a necessary step before producing ideal HCPs. Aromatic building blocks such as benzene, biphenyl and 1,3,5-triphenylbenzene have been commonly used to produce HCPs.^16,17,28^ Due to the absence of polar groups or heteroatoms, many reported HCPs, prepared from aromatic building blocks, lack strong CO_2_ binding sites and unfortunately tend to show a low or moderate CO_2_ adsorption.^16,18,19^ Heterocyclic compounds that consist of nitrogen, oxygen and sulfur have also been widely investigated in this regard.^12,20–22^ Attributed to the lone pair electrons of heteroatoms, which are playing a crucial role in offering interaction sites through dipole–dipole interactions,^23^ heterocyclic network could exhibit enhanced CO_2_ adsorption, and this value is similar to that of polymers generated from benzene, though the surface area is much lower than that of the later.^16^ Moreover, simulations based on employing density functional theory (DFT) calculations have further indicated that with the largest binding energy towards CO_2_, more negative charge distribution over moieties of a precursor could help in improving the interactions with CO_2_ molecules.^24^ So it is not hard to speculate that, apart from aromatic building blocks and heterocyclic compounds, the building units rich in electron are still probably better choices to produce HCPs with high CO_2_ capture capacity.

Polycyclic aromatic hydrocarbons (PAHs) are a big family of organic compounds with two or more laterally fused benzene rings and produced by combustion processes involving carbonaceous fuels. Owing to the structurally composed π conjugated systems, they are rich in electron and more likely to be promising building blocks for creating novel HCPs.^25^ However, PAHs have gathered growing concerns, because they are widely distributed environmental containment, do not degrade easily under natural conditions and often have detrimental biological effects, toxicity, mutagenicity and carcinogenicity.^26,27^ Considering their ubiquitous occurrence in all components of environment, recalcitrance, bioaccumulation potential and carcinogenic activity, controlling the emission of PAHs has become more urgent and far-reaching.^26,27^

What is more, it should be noted that, for the benefit of fabricating original HCPs with fascinating structures and multifunctional applications, a series of synthetic approaches such as the solvent knitting method,^28^ the knitting method with FDA as external crosslinker^16^ and the Scholl coupling reaction^17^ have been creatively reported. While in these strategies, the latest solvent knitting method, based on the Friedel–Crafts alkylation reaction mechanism, concerning the employment of dichloroalkane as both economical solvent and external crosslinker, was recently put forward by our group. Apart from distinguishing characteristics as simple, one-step, cost-effective, it is also something of an innovation in the constraint of methodology with respect to providing a wealth of splendid opportunities for polymer networks with higher surface area, narrow pore size distribution, good gas adsorption performance and so on, thus being considered to be a reliable polymerization technique.^28^

We, therefore, set out to use low cost PAHs such as fluoranthene, binaphthalene, naphthalene and phenanthrene aim to knit microporous organic polymers for efficient gas adsorption. The fluoranthene-based polymer of all the four synthesized samples reveals the highest surface area of 1788 m^2^ g^−1^ and the best CO_2_ uptake property of 24.79 wt% (273.15 K/1.00 bar), H_2_ storage ability of 2.20 wt% (77.3 K/1.13 bar) and CH_4_ adsorption capacity of 2.79 wt% (273.15 K/1.00 bar). In addition, it is noteworthy that the electron-rich PAHs have been found to be novel building blocks for the production of microporous polymers with high gas adsorption properties. The most plausible reasons for the distinct structural properties and higher gas uptake capacity of these polymers may largely be attributed to their porous nature including the pore size, pore size distribution as well as BET surface area, which is expected to give more insight to produce microporous with desirable properties.

## Experimental section

### Materials

The starting chemicals such as fluoranthene, binaphthalene, naphthalene and phenanthrene were purchased from Aladdin Chemical Reagent Corporation (Shanghai, China) and used as received. Dichloromethane (CH_2_Cl_2_), ethanol, hydrochloride (HCl) and anhydrous aluminum chloride (AlCl_3_) were obtained from Sinopharm Chemical Reagent Limited Company (Shanghai, China) and also used as received. Unless otherwise specified, all the available reagents were acquired from commercial suppliers and used without any further purification.

### Synthesis of Polymer 1

Under a nitrogen atmosphere, fluoranthene (2 mmol, 0.404 g) was dispersed in CH_2_Cl_2_ (8 mL) for about 30 min, and then anhydrous AlCl_3_ (24 mmol, 3.204 g) was added to the solution. The mixture was allowed to react at 20 °C for 4 h, 30 °C for 8 h, 40 °C for 12 h, 60 °C for 12 h and 80 °C for 24 h by vigorously stirring. After cooling to room temperature, the solid product was quenched by using 20 mL HCl–H_2_O (v/v = 2 : 1), washed several times with deionized water and ethanol respectively, further purified by Soxhlet extractor with ethanol for 24 h, and finally dried in a vacuum oven at 70 °C for 48 h. The synthesized material was obtained as a black solid. Yield: 147%.

### Synthesis of Polymer 2

This material was synthesized by employing the same method as described above for Polymer 1 and prepared by treating binaphthalene (1 mmol, 0.254 g) with anhydrous AlCl_3_ (32 mmol, 4.272 g) in CH_2_Cl_2_ (8 mL). The material was obtained as a black solid. Yield: 123%.

### Synthesis of Polymer 3

This material was synthesized by employing the same method as described above for Polymer 1 and prepared by treating naphthalene (4 mmol, 0.512 g) with anhydrous AlCl_3_ (64 mmol, 8.544 g) in CH_2_Cl_2_ (8 mL). The material was obtained as a black solid. Yield: 132%.

### Synthesis of Polymer 4

This material was also synthesized by employing the same method as described above for Polymer 1 and prepared by treating phenanthrene (3 mmol, 0.534 g) with anhydrous AlCl_3_ (72 mmol, 9.612 g) in CH_2_Cl_2_ (8 mL). The material was obtained as a black solid. Yield: 110%.

### FT-IR and solid NMR experiments

FT-IR spectra were recorded by a Bruker VERTEX 70 FT-IR spectrometer employing the KBr disk method. Solid state ^13^C cross-polarization/magic-angle spinning nuclear magnetic resonance (^13^C CP/MAS NMR) spectra were performed on a WB 400 MHz Bruker Avance II spectrometer, and collected with a spinning rate of 20 kHz by using a 2.5 mm double-resonance MAS probe.

### FE-SEM, TEM and TGA analyses

The field-emission scanning electron microscopy (FE-SEM) images were recorded by employing an FEI Sirion 200 field-emission scanning electron microscope operating at 10 kV. Before measurement, all samples were dried in a vacuum oven at 70 °C for 24 h and then sputter coated with platinum. The transmission electron microscopy (TEM) images were recorded on a Tecnai G2 F30 microscope (FEI Corp. Holland) operating at 200 kV. Thermogravimetric analysis (TGA) was performed from room temperature to 850 °C under a nitrogen atmosphere, by employing a Perkin Elmer Instrument Pyris1 TGA with a heating rate of 10 °C min^−1^.

### Gas adsorption measurements

All the four samples were degassed for a minimum of 8 h at 120 °C under vacuum of 10^−5^ bar prior to the measurement of their gas adsorption properties. Gas (N_2_, CO_2_, H_2_ and CH_4_) sorption properties and specific surface area of samples were measured by a Micromeritics ASAP 2020 surface area and porosity analyzer respectively. Pore size distribution was calculated by N_2_ adsorption isotherms using a Tarazona nonlocal density functional theory (NLDFT) model assuming slit pore geometry. Total pore volumes (*V*_total_) were derived from nitrogen sorption isotherms at relative pressure *P*/*P*_0_ = 0.995.

## Results and discussion

**Scheme 1:**
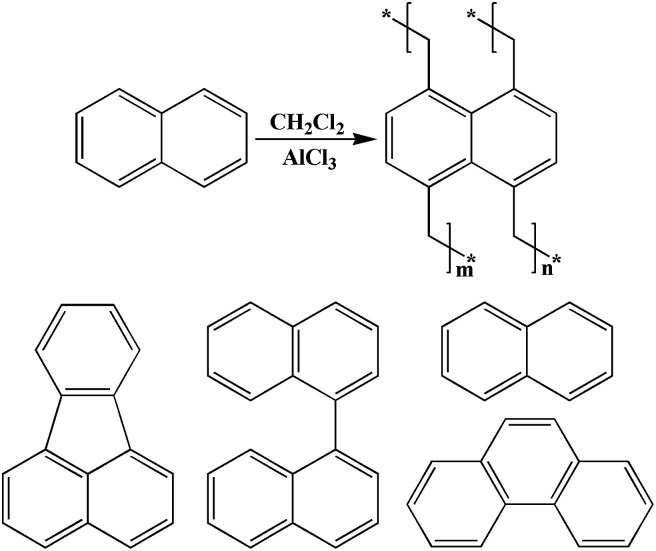
The proposed synthetic pathway to the network structures and building blocks.

**Fig. 1:**
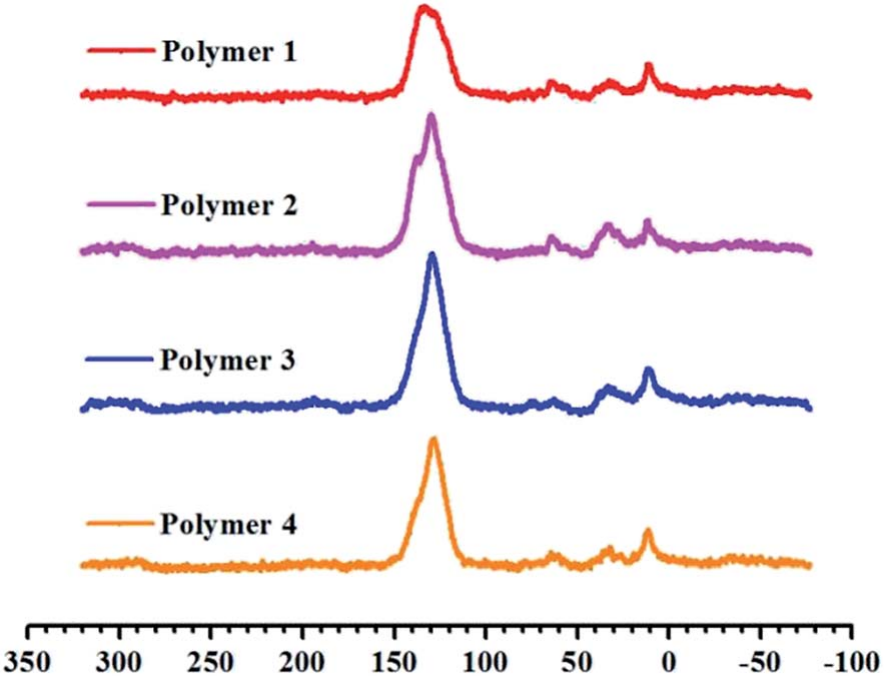
Cross-polarization (CP) ^13^C MAS natural abundance NMR spectra of materials from Polymer 1 to Polymer 4.

Scheme 1 outlines the proposed synthetic routes and initial building blocks such as fluoranthene, binaphthalene, naphthalene and phenanthrene for the four polymers. The synthesized polymers were then structurally characterized by FT-IR and solid state ^13^C CP/MAS NMR after their purification. As shown in Fig. S6–S9,† the C–H stretching vibrations are directly observed near 2920 cm^−1^, which firmly confirm the presence of methylene groups in chemical structure of these polymers, while Fig. S2–S5† show the basic FT-IR details of the four monomers. The solid state ^13^C CP/MAS NMR (Fig. 1) spectra show various types of carbon signals. For example, the resonance peaks near 130 ppm and 137 ppm are due to the non-substituted aromatic carbon and substituted aromatic carbon respectively, while the resonance peaks near 37 ppm are primarily attributed to the methylene carbon, which once again clearly indicate the formation of methylene linked polymers that originate from the use of CH_2_Cl_2_ as both economical solvent and external crosslinker. Taken together, both the FT-IR and ^13^C CP/MAS NMR results convincingly proved that these PAH-based microporous organic polymers were successfully produced by simple one-step Friedel–Crafts alkylation reaction.

**Fig. 2:**
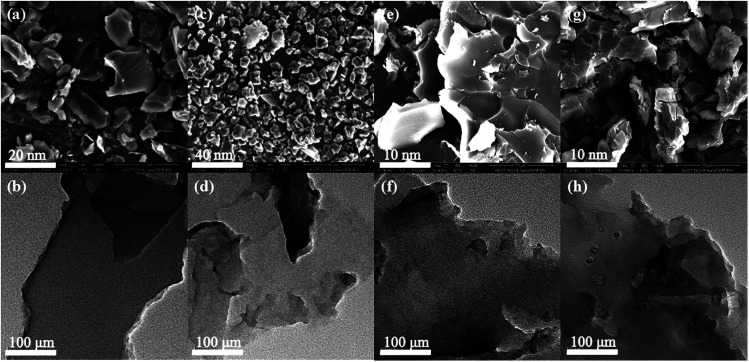
The FE-SEM image (a) and TEM image (b) of Polymer 1; the FE-SEM image (c) and TEM image (d) of Polymer 2; the FE-SEM image (e) and TEM image (f) of Polymer 3; the FE-SEM image (g) and TEM image (h) of Polymer 4.

The surface morphologies and inherent porous structures of all polymers were examined by electron microscopy. Fig. 2 shows the FE-SEM images of Polymer 1 to Polymer 4. All four polymer samples are irregular blocks without any long-range order, and in comparison, Fig. S1† gives the FE-SEM images of corresponding building units at different magnifications. Fig. 2 also shows the TEM images of Polymer 1 to Polymer 4 at the same magnification. All four polymer samples have hierarchically porous structure with abundant micropores and a small amount of mesopores. The TGA analysis curves in Fig. S10† indicated that all the four polymer networks have similar decomposition behaviors except Polymer 1 in a high temperature region when the temperature is more than 450 °C, which is mainly caused by different crosslinking degree of the networks. The significant weight loss in a high temperature range over 400 °C under a nitrogen atmosphere is the result of the destruction of polymeric networks, which importantly verifies the good chemical and thermal stability of these polymers.

**Fig. 3:**
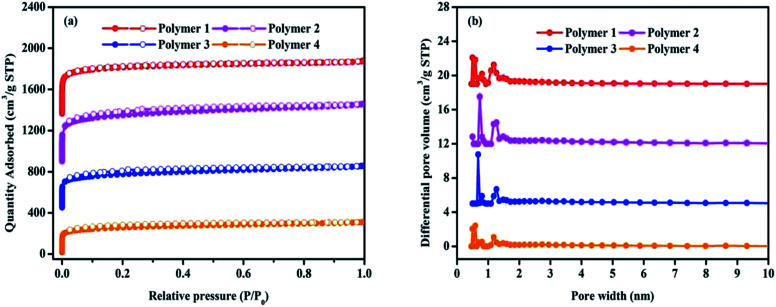
Nitrogen adsorption and desorption isotherms at 77.3 K (a) and pore distribution of pore size distribution calculated using DFT methods (slit pore models, differential pore volumes). Pore width (b) of samples.

The porosity of polymers were determined by nitrogen adsorption and desorption isotherms at 77.3 K. As shown in Fig. 3a, all the adsorption isotherms exhibited a type I character with a steep nitrogen gas uptake at low relative pressure (*P*/*P*_0_ < 0.001), implying the adsorption into abundant micropores, which is in good agreement with the results of TEM. It is fairly obvious that all the microporous networks are accompanied with primary micropores, and bits of mesopores that are reflected by almost no obvious hysteresis loop in the middle pressure area. In Fig. 3b, the pore size distribution with a large pore volume calculated by the NLDFT model indicated that all the four polymers possess hierarchical pore distribution, and the pore size distribution principally covers a very narrow range no more than 2 nm in microporous religion, including the ultramicropore whose size is less than 0.7 nm in diameter. Table 1 presents more details about these polymers that the total pore volumes vary between 0.48 cm^3^ g^−1^ and 0.86 cm^3^ g^−1^ with micropore volume from 0.27 cm^3^ g^−1^ to 0.53 cm^3^ g^−1^ as well as the *t*-plot microporous area over BET surface area is within the scope of 65.57% to 75.89%. Based on the above analysis, it can be concluded that the solvent knitting method could bridge various building units to generate microporous organic materials under mild conditions.

**Table 1:** Porosity properties of the polymer networks

Samples	*S*_BET_ a (m^2^ g^−1^)	*S*_L_ b (m^2^ g^−1^)	MA^c^ (m^2^ g^−1^)	PV^d^ (cm^3^ g^−1^)	MPV^e^ (cm^3^ g^−1^)	MA^f^ (%)
Polymer 1	1788	2102	1357	0.82	0.53	75.89
Polymer 2	1702	2063	1116	0.86	0.44	65.57
Polymer 3	1227	1490	807	0.63	0.32	65.77
Polymer 4	978	1173	684	0.48	0.27	69.94

a Apparent surface area calculated from nitrogen adsorption isotherms at 77.3 K using the BET equation.

b Surface area calculated from nitrogen adsorption isotherms at 77.3 K using the Langmuir equation.

c *t*-Plot micropore area.

d Pore volume calculated from the nitrogen isotherms at *P*/*P*_*0*_ = 0.995 and 77.3 K.

e *t*-Plot micropore volume calculated from the nitrogen isotherms at *P*/*P*_*0*_ = 0.050.

f *t*-Plot microporous area/BET surface area × 100%.

**Fig. 4:**
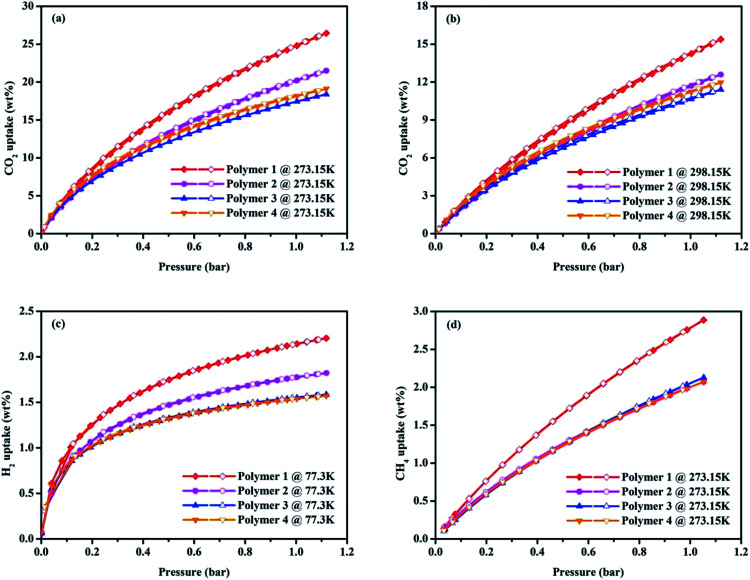
Volumetric CO_2_ adsorption isotherms and desorption isotherms up to 1.13 bar at 273.15 K (a), volumetric CO_2_ adsorption isotherms and desorption isotherms up to 1.13 bar at 298.15 K (b), volumetric H_2_ adsorption isotherms and desorption isotherms up to 1.13 bar at 77.3 K (c) and volumetric CH_4_ adsorption isotherms and desorption isotherms up to 1.13 bar at 273.15 K (d) of all the samples.

The interesting physical properties of these knitted polymers, as listed in Table 1, including their high surface area and microporous nature prompted us to pay more attention to their gas uptake performance. Taking together the CO_2_ isotherms in Fig. 4a and the gas uptake capacities in Table 2, it is obvious Polymer 1 has an ultrahigh BET surface area of 1788 m^2^ g^−1^ and the highest CO_2_ adsorption amount of 24.79 wt% (273.15 K/1.00 bar) compared to the Polymer 2, Polymer 3 and Polymer 4 whose BET surface areas are 1702 m^2^ g^−1^, 1227 m^2^ g^−1^ and 978 m^2^ g^−1^ with corresponding CO_2_ uptake capacity of 20.19 wt%, 17.44 wt% and 18.15 wt% (273.15 K/1.00 bar) respectively. It may be due to their much higher microporosity and ultramicropore whose diameter is comparable to the kinetic diameter of CO_2_ thus increasing the interaction between CO_2_ molecules and the pore walls.^29^ Although lower than the reported imine-linked porous polymer network PPF-1 (26.7 wt%, *S*_BET_ = 1740 m^2^ g^−1^),^30^ the CO_2_ uptake of Polymer 1 is comparable with some reported porous polymers with high CO_2_ uptake performance such as carbazolic porous organic framework Cz-POF-3 (4.77 mmol g^−1^, *S*_BET_ = 1927 m^2^ g^−1^),^31^ benzimidazole-linked polymer BILP-4 (5.3 mmol g^−1^, *S*_BET_ = 1235 m^2^ g^−1^),^32^ azo-linked porous organic polymer ALP-1 (5.37 mmol g^−1^, *S*_BET_ = 1235 m^2^ g^−1^),^33^ fluorinated covalent triazine-based framework FCTF-1-600 (5.53 mmol g^−1^, *S*_BET_ = 1535 m^2^ g^−1^),^34^ and bipyridine-based nitrogen rich covalent triazine framework bipy-CTF600 (5.58 mmol g^−1^, *S*_BET_ = 2479 m^2^ g^−1^).^35^ The CO_2_ capture capacity of Polymer 1 is higher than many CMPs produced by Sonogashira–Hagihara coupling reactions such as the pyrene-based porous aromatic framework of PAF-26 (1.16 mmol g^−1^, *S*_BET_ = 702 m^2^ g^−1^),^36^ the amide-functionalized CMP of CMP-1-AMD1 (1.51 mmol g^−1^, *S*_BET_ = 316 m^2^ g^−1^),^37^ the hexabenzocoronene-based porous organic polymers of HBC-POP-1 (2.05 mmol g^−1^, *S*_BET_ = 668 m^2^ g^−1^),^38^ the tri(4-ethynylphenyl)amine-based porous aromatic framework of PAF-34 (2.50 mmol g^−1^, *S*_BET_ = 953 m^2^ g^−1^)^39^ and the post-metalation of the porous aromatic framework of PAF-26-COOMg (2.85 mmol g^−1^, *S*_BET_ = 572 m^2^ g^−1^)^40^ at 273.15 K/1.00 bar. Moreover, the capture of CO_2_ in Polymer 1 is also much higher than some types of porous materials with much higher BET surface area under the similar conditions such as the tetraphenylmethane-based HCP (1.66 mmol g^−1^, *S*_BET_ = 1679 m^2^ g^−1^),^41^ COF-102 (1.56 mmol g^−1^, *S*_BET_ = 3620 m^2^ g^−1^)^42^ and PAF-1 (2.1 mmol g^−1^, *S*_BET_ = 5460 m^2^ g^−1^).^43^ In order to determine the binding affinity between the polymers and CO_2_ molecules, the isosteric heat (Qst) of the four obtained polymers toward CO_2_ was calculated from the adsorption isotherms at 273.15 K and 298.15 K using the Clausius–Clapeyron equation (Fig. S11†),^44^ which were found to be of 25.65–24.41 kJ mol^−1^, 26.40–24.82 kJ mol^−1^, 26.93–25.30 kJ mol^−1^ and 28.09–26.38 kJ mol^−1^ for Polymer 1, Polymer 2, Polymer 3 and Polymer 4 respectively.

**Table 2:** Gas uptake values of the polymer networks

Samples	CO_2_ uptake^a^ (wt%)	CO_2_ uptake^b^ (wt%)	CH_4_ uptake^c^ (wt%)	H_2_ uptake^d^ (wt%)
Polymer 1	24.79	14.79	2.79	2.20
Polymer 2	20.19	11.69	2.00	1.82
Polymer 3	17.44	10.66	2.08	1.59
Polymer 4	18.15	11.23	2.00	1.57

a CO_2_ uptake determined volumetrically using a Micromeritics ASAP 2020 M analyzer at 1.00 bar and 273.15 K.

b CO_2_ uptake determined volumetrically using a Micromeritics ASAP 2020 M analyzer at 1.00 bar and 298.15 K.

c CH_4_ uptake determined volumetrically using a Micromeritics ASAP 2020 M analyzer at 1.00 bar and 273.15 K.

d H_2_ uptake determined volumetrically using a Micromeritics ASAP 2020 M analyzer at 1.13 bar and 77.3 K.

As the increasing demand for energy and environmental concerns has made H_2_ storage much appealing in porous materials research, investigation of H_2_ uptake performance of polymers has become more and more attractive. As shown in Fig. 4c, the hydrogen adsorption and desorption measurements at low pressure were carried out at 77.3 K, and all the isotherms of polymers for H_2_ adsorption are fully reversible. Intriguingly, Polymer 1 has the highest H_2_ uptake of 2.20 wt% (77.3 K/1.13 bar) compared to the Polymer 2, Polymer 3 and Polymer 4 whose H_2_ uptake capacities are 1.82 wt%, 1.59 wt% and 1.57 wt% respectively under the similar conditions. Though lower than that of the CPOP-1 (2.80 wt%),^45^ zeolite-like Carbon (2.60 wt%),^46^ MIL-101(2.50 wt%)^47^ and carbon AX-21 (2.40 wt%),^48^ the H_2_ adsorption ability of Polymer 1 is still comparable to SPOP-3 (2.22 wt%)^49^ and PAF-3 (about 2.07 wt%)^43^ at 77.3 K/1.00 bar, and also much higher than that of many reported materials such as benzene-based CMP of CMP-0 (1.4 wt%),^45^ the nanoporous organic framework of NPOF-2 (1.45 wt%)^50^ as well as the tetraphenylethylene-based copolymer networks (0.95–1.76 wt%)^51^ under similar conditions.

Touted as an ideal energy resource, methane storage may be a serious issue in future energy schemes, so the methane storage capacity of the polymers is also worth exploring. As shown in Fig. 4d, Polymer 1 has a better methane adsorption of 2.79 wt% (273.15 K/1.00 bar) than that of Polymer 2, Polymer 3 and Polymer 4 whose methane storage abilities are 2.00 wt%, 2.08 wt% and 2.00 wt% (273.15 K/1.00 bar) respectively. It is easy to note that the methane storage of Polymer 1 is, though lower than the carbonized FCDTPA-K-700 (2.36 mmol g^−1^ at 273.15 K/1.13 bar),^15^ the activated carbon of K-PAF-1-600 (2.4 mmol g^−1^ at 273.15 K/1.00 bar) that stemmed from PAF-1,^52^ while still much higher than that of the reported FCDTPA (0.89 mmol g^−1^ at 273.15 K/1.13 bar),^15^ and the solution-processable hypercrosslinked polymers (0.08–0.14 wt% at 273.15 K/1.13 bar).^53^

Keeping in view different structure and gas capture ability of these polymers, the correlation of structural diversity and gas adsorption performance has come up as an interesting phenomenon. Various efforts have been made to seek for the best interpretation for such abnormal phenomenon, and in the most of cases it has been ascribed to the porous nature of such materials including the pore size and pore size distribution as well as surface area of polymers. Universally it is acknowledged that factors like slight variations in the chemical structure, aggregation structure, and porous properties including the specific surface area, micropore volume, pore shape, pore size and pore size distribution largely affect the CO_2_ adsorption capacity of microporous organic polymers.^54^ However, more convincing explanation can be directly derived from Fig. S12.† As evident, based on some selected microporous hypercrosslinked polymers,^16,28^ the relationship of BET surface area and CO_2_ uptake at 273.15 K/1.00 bar is vividly presented in a good fitting equation of *y* = 0.00676*x* + 3.8732 with correlation coefficient value *R*^2^ = 0.99728. According to the experimental results, the obtained polymers with corresponding surface area and CO_2_ uptake at 273.15 K/1.00 bar have been totally scattered at different places above the equation. So it is not hard to understand that, aside from the BET surface area and other possible factors like the chemical structure of monomers, aggregation structure, micropore volume, pore shape, the pore size and pore size distribution are extremely important for CO_2_ adsorption. The previous reports have indicated that the pore size less than 1 nm is useful to adsorb CO_2_ molecules,^55^ especially the ultramicropore with diameter no more than 0.7 nm, makes marvelous contributions to CO_2_ uptake, for the small pore size could favorably increase the interaction between the CO_2_ molecules and the pore walls of polymers.^29^ Notably, the PAHs with available π conjugated systems have obvious superiority in electron distribution, which have emerged as a superior class of versatile building blocks for establishing microporous organic polymers with high surface area and enhancing gas uptake ability. Furthermore, this finding not only largely gives some enlightenment on the rational design and synthesis of desirable polymers, but may also positively facilitate the considerable development of HCPs in many interdisciplinary domains in the future.

## Conclusions

In summary, four PAH-based microporous organic polymers were synthesized by the solvent knitting method. As a matter of fact, Polymer 1 with an ultrahigh BET surface area of 1788 m^2^ g^−1^ displays the highest CO_2_ uptake capacity of 24.79 wt% (273.15 K/1.00 bar), H_2_ storage of 2.20 wt% (77.3 K/1.13 bar) and CH_4_ uptake performance of 2.79 wt% (273.15 K/1.00 bar) among these polymers. Insights into the porous nature of polymers will be much helpful in building polymers with high surface area for outstanding gas adsorption. More importantly, the electron-rich PAHs, containing π conjugated systems, have been found to be novel building blocks for the formation of polymers, which inspires the following research upon the rational design and synthesis of desired polymer networks. Given the merits as low cost, high surface area, efficient CO_2_ absorption performance as well as H_2_ uptake and CH_4_ storage capacity, and high thermal stability, these PAH-based polymers are considered to be good candidates for the efficient separation of CO_2_ and energy storage materials under the optimized capture conditions.

## Conflicts of interest

There are no conflicts to declare.

## Supplementary Material

RA-008-C8RA01332B-s001
