# Supplementary material for: Knitting polycyclic aromatic hydrocarbon-based microporous organic polymers for efficient CO2 capture
Source: RSC Adv. 2018 Mar 13;8(19):10347–54. doi: 10.1039/c8ra01332b (PMC9078886; doi:10.1039/c8ra01332b)
Supplement: RA-008-C8RA01332B-s001 [file RA-008-C8RA01332B-s001.pdf]

Electronic Supplementary Material (ESI) for RSC Advances.
This journal is © The Royal Society of Chemistry 2018

Supporting Information

Knitting polycyclic aromatic hydrocarbon-based microporous organic polymers for efficient CO_2 capture

Shuangshuang Hou,[a] Shaolei Wang,[a] Xuejun Long,[b] and Bien Tan*[a]

[a] Key Laboratory for Large-Format Battery Materials and System, Ministry of Education. Hubei Key Laboratory of Material Chemistry and Service Failure. School of Chemistry and Chemical Engineering, Huazhong University of Science and Technology, Wuhan 430074, P. R. China. Tel: +86-27-87558172; Fax: +86-27-87543632; E-mail: *bien.tan@mail.hust.edu.cn (Tan B)*

[b] Engineering Research Center for Clean Production of Textile Printing and Dyeing, Ministry of Education, Wuhan Textile University, Wuhan 430073, P. R. China.

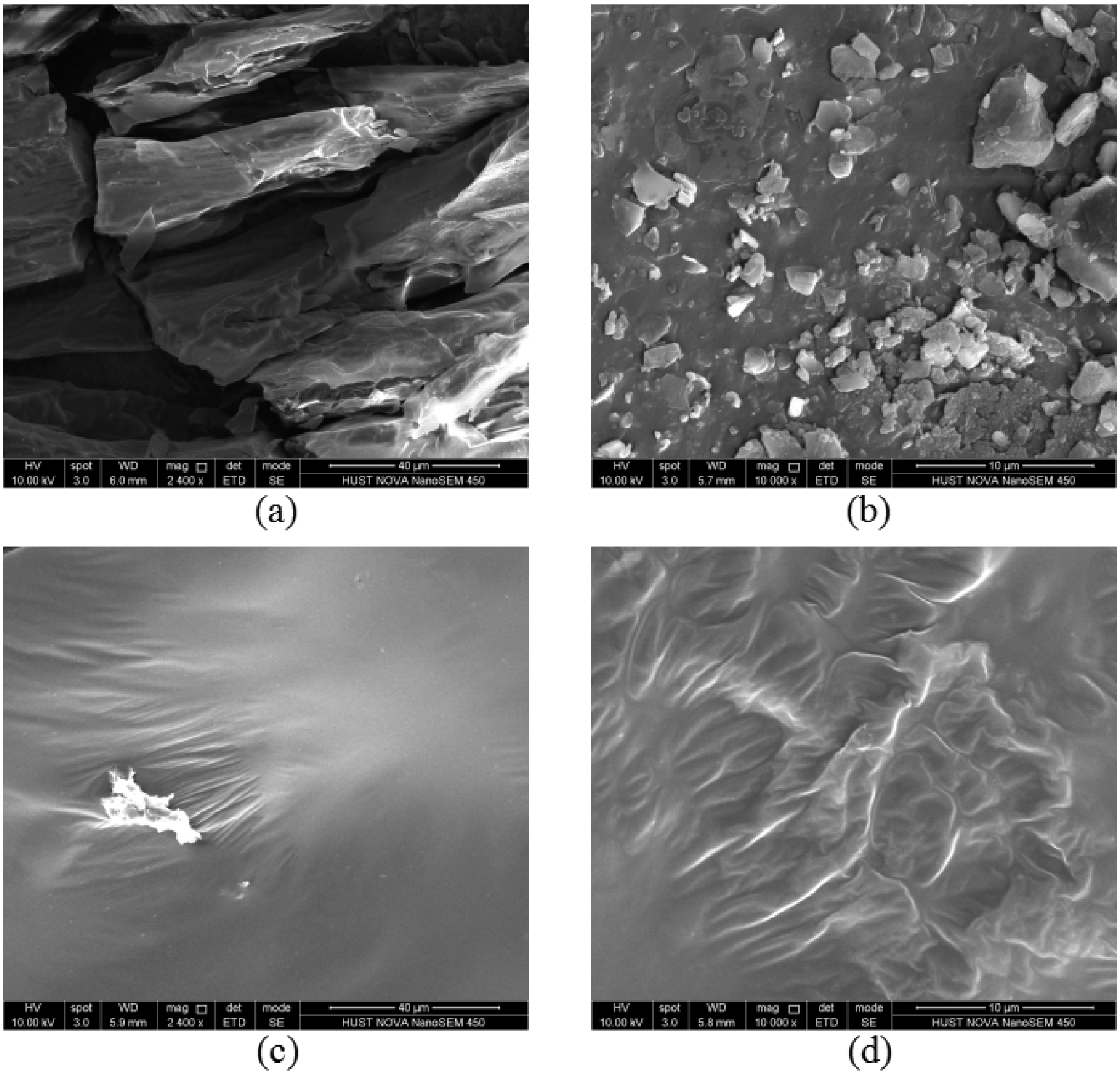

Figure S1. SEM image (a) of fluoranthene; SEM image (b) of binaphthalene; SEM image (c) of naphthalene; SEM image (d) of phenanthrene at different scale bars.

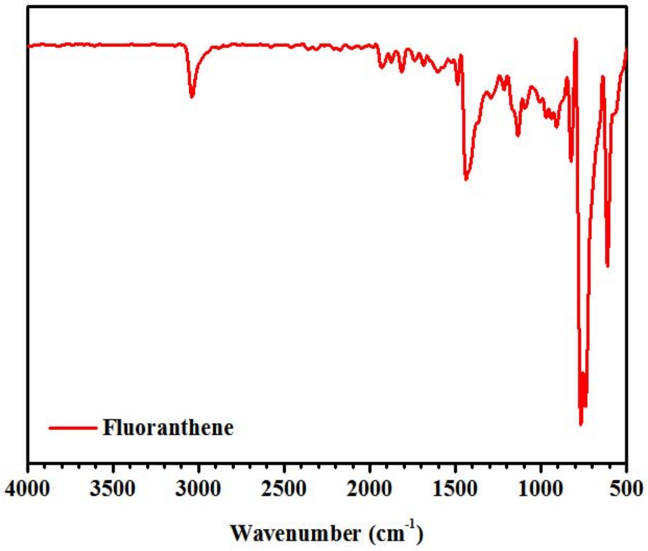

Figure S2. Fourier transform infrared spectrum of fluoranthene.

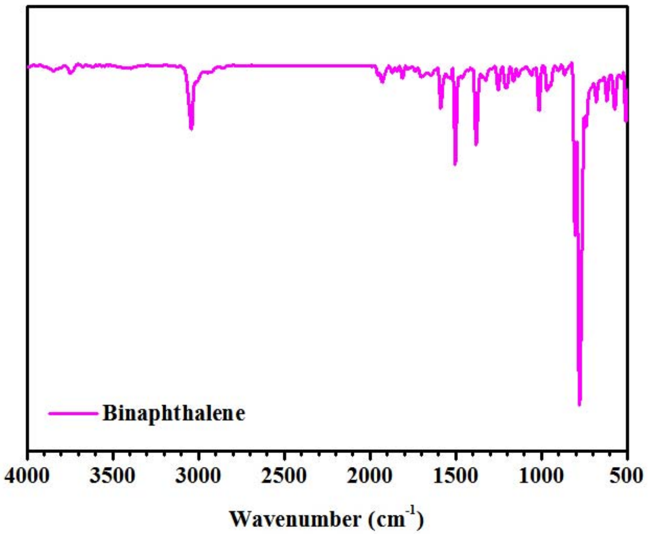

Figure S3. Fourier transform infrared spectrum of binaphthalene.

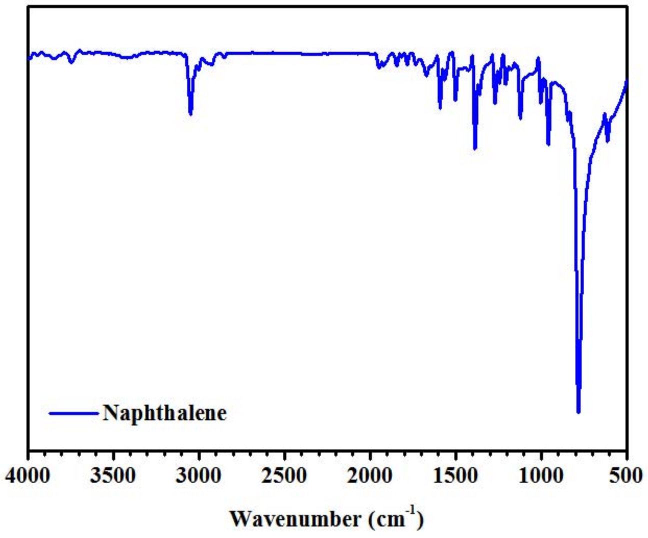

Figure S4. Fourier transform infrared spectrum of naphthalene.

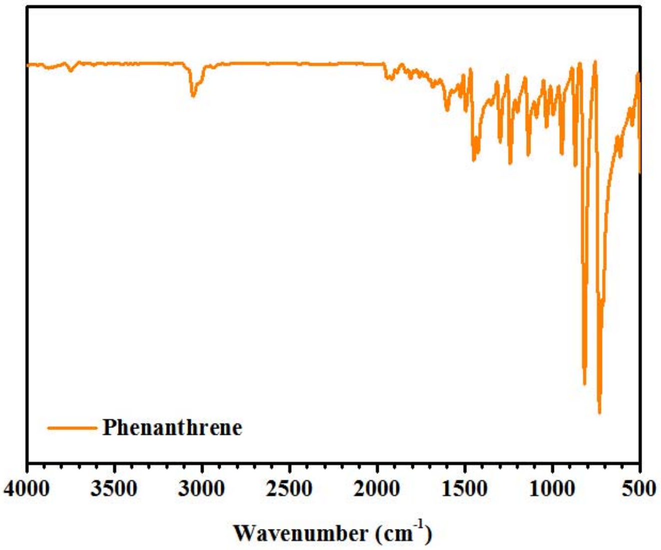

Figure S5. Fourier transform infrared spectrum of phenanthrene.

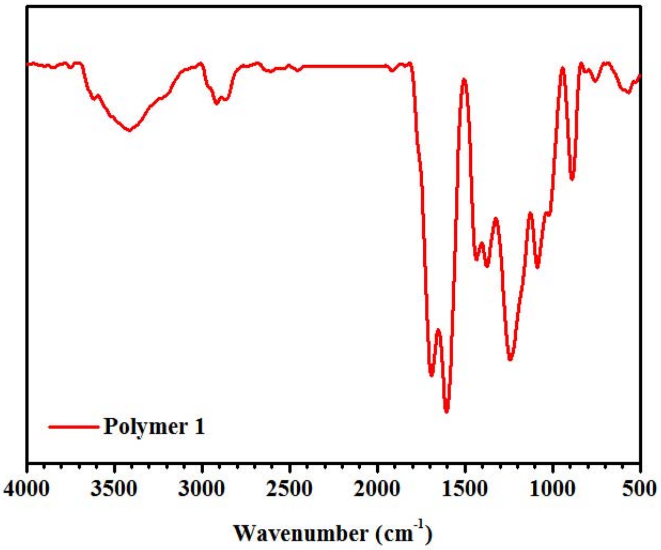

Figure S6. Fourier transform infrared spectrum of **Polymer 1**.

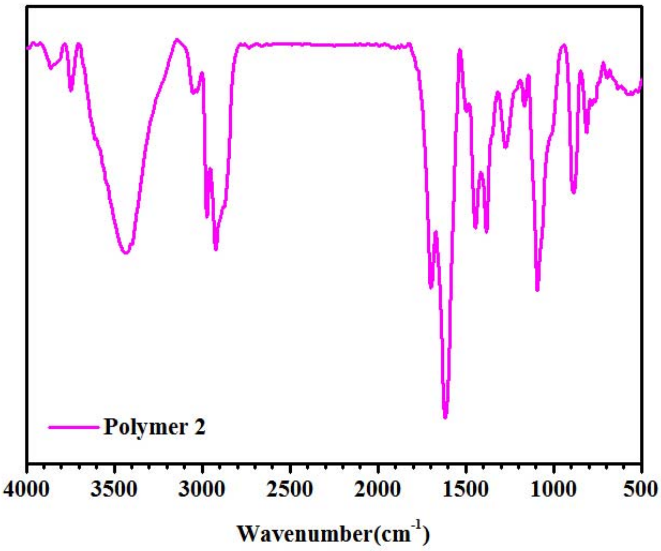

Figure S7. Fourier transform infrared spectrum of **Polymer 2**.

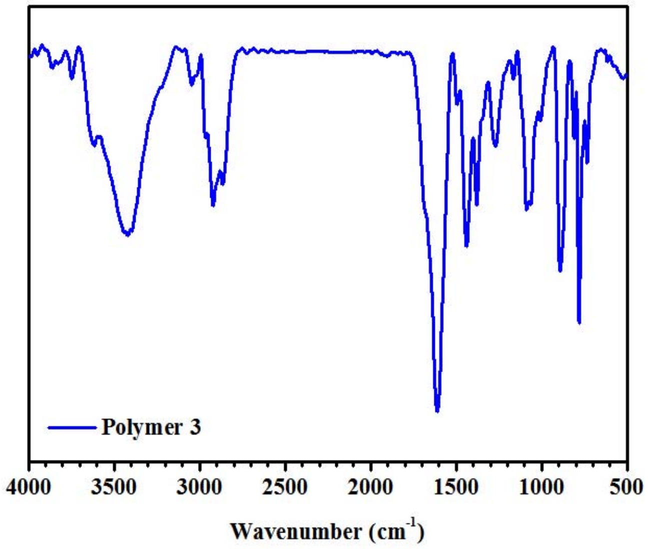

Figure S8. Fourier transform infrared spectrum of **Polymer 3**.

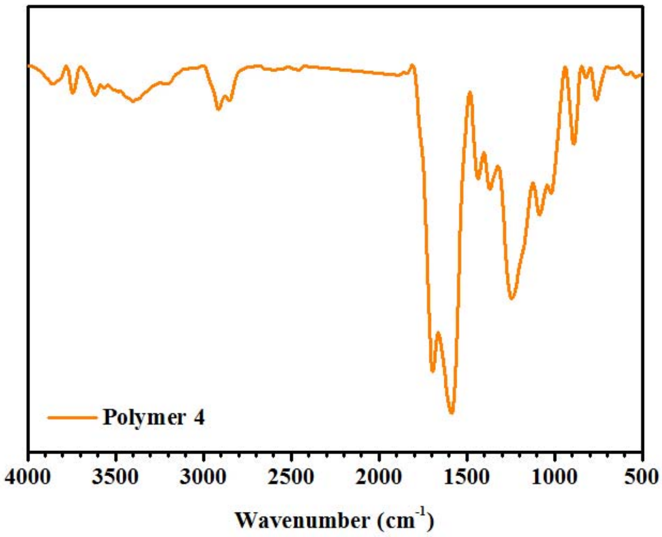

Figure S9. Fourier transform infrared spectrum of **Polymer 4**.

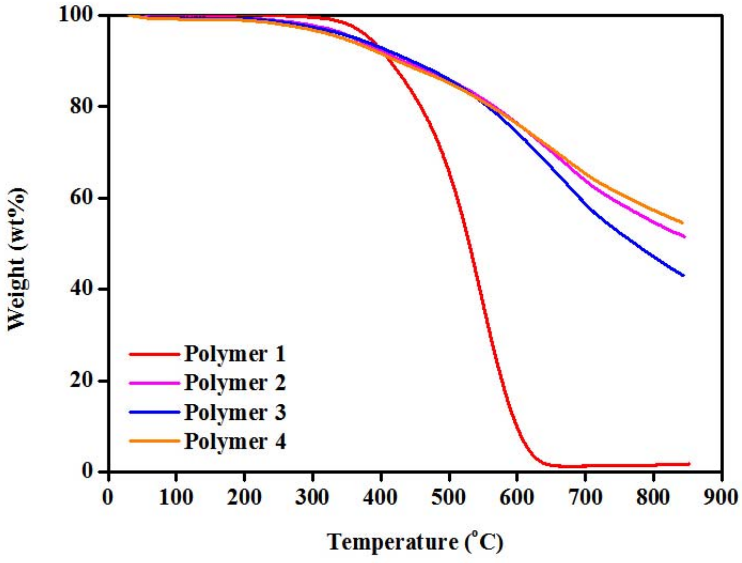

Figure S10. Thermogravimetric analysis of materials from **Polymer 1** to **Polymer 4**.

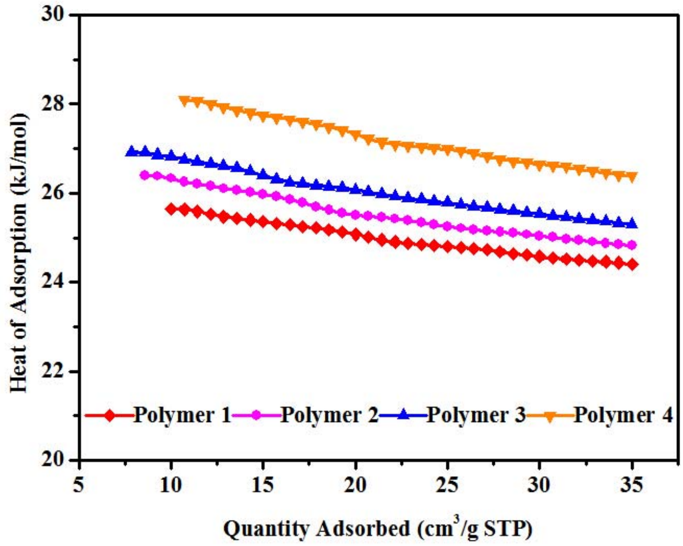

Figure S11. Isosteric heat of adsorption for CO_2 at different CO_2 loadings.

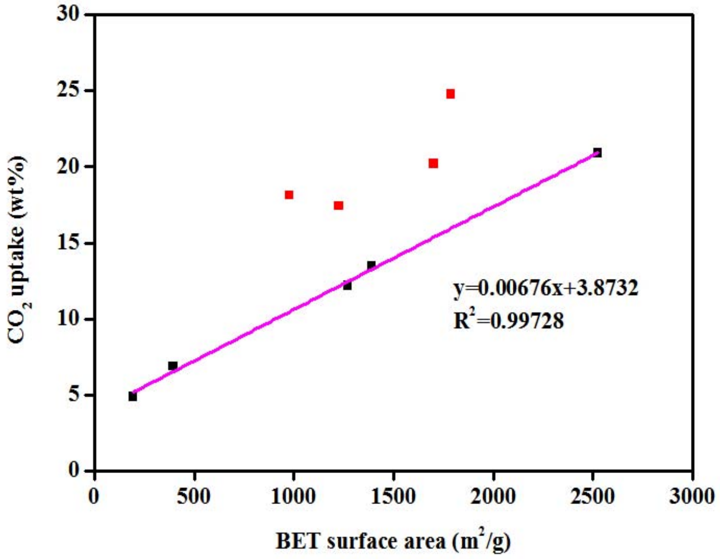

Figure S12. The fitting relationship of CO_2 uptake at 273.15 K/1.00 bar and BET surface area based on some selected hypercrosslinked polymers.

$$\textbf{Yield \%}=\frac{\mathbf{m_1(g)}}{\mathbf{m_2(g)}}\ \textbf{X 100\%}$$

Where m_1 is the weight of **Polymer 1**, **Polymer 2**, **Polymer 3** and **Polymer 4** measured respectively after drying in a vacuum oven at 70 °C for 24 h, m_2 is the weight of fluoranthene, binaphthalene, naphthalene and phenanthrene correspondingly.

Equation S1 The yield estimation of materials from **Polymer 1** to **Polymer 4**.
